# Super-resolution deep learning reconstruction to improve image quality of coronary CT angiography

**DOI:** 10.1093/radadv/umae001

**Published:** 2024-03-19

**Authors:** Nobuo Tomizawa, Yui Nozaki, Hideyuki Sato, Yuko Kawaguchi, Ayako Kudo, Daigo Takahashi, Kazuhisa Takamura, Makoto Hiki, Shinichiro Fujimoto, Iwao Okai, Seiji Koga, Shinya Okazaki, Kanako K Kumamaru, Tohru Minamino, Shigeki Aoki

**Affiliations:** Department of Radiology, Juntendo University Graduate School of Medicine, Tokyo 113-8421, Japan; Department of Cardiovascular Biology and Medicine, Juntendo University Graduate School of Medicine, Tokyo 113-8421, Japan; Department of Radiology, Juntendo University Graduate School of Medicine, Tokyo 113-8421, Japan; Department of Cardiovascular Biology and Medicine, Juntendo University Graduate School of Medicine, Tokyo 113-8421, Japan; Department of Cardiovascular Biology and Medicine, Juntendo University Graduate School of Medicine, Tokyo 113-8421, Japan; Department of Cardiovascular Biology and Medicine, Juntendo University Graduate School of Medicine, Tokyo 113-8421, Japan; Department of Cardiovascular Biology and Medicine, Juntendo University Graduate School of Medicine, Tokyo 113-8421, Japan; Department of Cardiovascular Biology and Medicine, Juntendo University Graduate School of Medicine, Tokyo 113-8421, Japan; Department of Cardiovascular Biology and Medicine, Juntendo University Graduate School of Medicine, Tokyo 113-8421, Japan; Department of Cardiovascular Biology and Medicine, Juntendo University Graduate School of Medicine, Tokyo 113-8421, Japan; Department of Cardiovascular Biology and Medicine, Juntendo University Graduate School of Medicine, Tokyo 113-8421, Japan; Department of Cardiovascular Biology and Medicine, Juntendo University Graduate School of Medicine, Tokyo 113-8421, Japan; Department of Radiology, Juntendo University Graduate School of Medicine, Tokyo 113-8421, Japan; Department of Cardiovascular Biology and Medicine, Juntendo University Graduate School of Medicine, Tokyo 113-8421, Japan; Department of Radiology, Juntendo University Graduate School of Medicine, Tokyo 113-8421, Japan

**Keywords:** coronary artery, coronary stenosis, CT angiography, image quality, model-based iterative reconstruction, super-resolution deep learning reconstruction

## Abstract

**Purpose:**

To compare the objective and subjective image quality and diagnostic performance for coronary stenosis of normal-dose model-based iterative reconstruction and reduced-dose super-resolution deep learning reconstruction in coronary CT angiography.

**Materials and Methods:**

This single-center retrospective study included 52 patients (mean age, 68 years ± 10 [SD]; 41 men) who underwent serial coronary CT angiography and subsequent invasive coronary angiography between January and November 2022. The first 25 patients were scanned with a standard dose using model-based iterative reconstruction. The last 27 patients were scanned with a reduced dose using super-resolution deep learning reconstruction. Per-patient objective and subjective image qualities were compared. Diagnostic performance of model-based iterative reconstruction and super-resolution deep learning reconstruction to diagnose significant stenosis on coronary angiography was compared per-vessel using receiver operating characteristics curve analysis.

**Results:**

The median tube current of super-resolution deep learning reconstruction was lower than that of model-based iterative reconstruction (median [IQR], 890 mA [680, 900] vs. 900 mA [895, 900], *P* = 0.03). Image noise of super-resolution deep learning reconstruction was lower than that of model-based iterative reconstruction (14.6 Hounsfield units ± 1.3 vs. 22.7 Hounsfield units ± 4.4, *P* < .001). Super-resolution deep learning reconstruction improved the overall subjective image quality compared with model-based iterative reconstruction (median [IQR], 4 [3, 4] vs 3 [3, 3], *P* = .006). No difference in the area under the receiver operating characteristic curve in diagnosing coronary stenosis using super-resolution deep learning reconstruction (0.96; 95% CI, 0.92-0.99) and model-based iterative reconstruction (0.96; 95% CI, 0.92-0.98; *P* = .98) was observed.

**Conclusion:**

Our exploratory analysis suggests that super-resolution deep learning reconstruction could improve image quality with lower tube current settings than model-based iterative reconstruction with similar diagnostic performance to diagnose coronary stenosis in coronary CT angiography.

**Abbreviations**

CNR = contrast-to-noise ratio, ICC = intraclass correlation coefficient, MBIR = model-based iterative reconstruction, SNR = signal-to-noise ratio, SR-DLR = super-resolution deep learning reconstruction

**Summary**

Super-resolution deep learning reconstruction improved both objective and subjective image quality over model-based iterative reconstruction in the diagnosis of coronary artery stenosis, with similar diagnostic performance and reduced tube current.

**Key Results**

- Super-resolution deep learning reconstruction (SR-DLR) reduced image noise compared with model-based iterative reconstruction (MBIR) (14.6 HU ± 1.3 vs. 22.7 HU ± 4.4, *P* < .001).
- SR-DLR improved the subjective image quality compared with MBIR (median [IQR], 4 [3, 4] vs. 3 [3, 3], *P* = .006).
- The area under the receiver operating characteristic curve in diagnosing coronary stenosis using SR-DLR (0.96; 95% CI, 0.92-0.99) and MBIR (0.96; 95% CI, 0.92-0.98; *P* = .98) seem comparable.

## Introduction

Improvement of computing power has allowed advanced reconstruction methods for CT images. Iterative reconstruction, introduced more than a decade ago, has reduced image noise and reduced the radiation dose of CT scans.^1^ Initially, iterative reconstruction was combined with filtered back projection to save the computational cost. With the improvements in computer performance, model-based iterative reconstruction (MBIR) has become available.^2^ MBIR has been advantageous in coronary CT angiography because it improves spatial resolution while reducing image noise; however, it has the disadvantage of long reconstruction time.

Recently, deep learning reconstruction has become available. Unlike MBIR, this method could reduce image noise with a short reconstruction time.^3^ Furthermore, super-resolution deep learning reconstruction (SR-DLR) has been introduced to improve spatial resolution, which could be achieved by training the algorithm using data acquired from an ultra-high-resolution CT.^4^ Several studies have compared the image quality of SR-DLR with other reconstruction methods in coronary CT angiography, but these studies used the same raw data.^4–6^ A phantom study showed that SR-DLR could improve image noise and spatial resolution while reducing radiation dose compared with MBIR.^7^ Based on this study, we hypothesized that SR-DLR could improve image quality and reduce radiation dose compared with MBIR. The present exploratory study sought to compare the objective and subjective image quality and diagnostic performance of normal-dose MBIR and reduced-dose SR-DLR for coronary artery stenosis in coronary CT angiography.

## Materials and methods

### Patients

**Figure 1.**
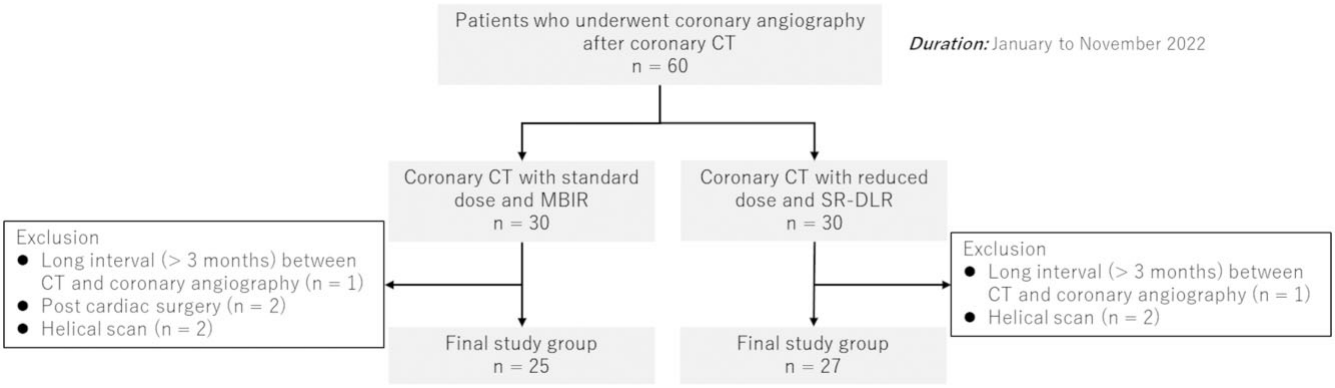
Patient flowchart. The study initially included 60 consecutive patients who underwent coronary angiography after coronary CT. After excluding ineligible patients, the standard-dose MBIR and reduced-dose SR-DLR groups included 25 and 27 patients, respectively. MBIR = model-based iterative reconstruction, SR-DLR = super-resolution deep learning reconstruction.

This single-center retrospective study was approved by the institutional review board, and the requirement for written informed consent was waived. The study included 60 patients who underwent coronary CT angiography with subsequent invasive coronary angiography between January and November 2022 (Figure 1). Exclusion criteria were as follows: interval between coronary CT angiography and invasive coronary angiography longer than 3 months (*n* = 2), after cardiac surgery (*n* = 2), and helical scan (*n* = 4). We excluded patients with helical scan because SR-DLR was only available for volume scan data. Based on a phantom study,^7^ the institutional protocol was modified in July 2022. The first half of the patients (*n* = 25) was scanned at normal dose and reconstructed with MBIR, whereas the second half (*n* = 27) was scanned at reduced dose and reconstructed with SR-DLR. Patient data were obtained from medical records.

### Coronary CT angiography

A 320-row CT was used to perform coronary CT angiography (Aquilion ONE Prism Edition; Canon Medical Systems). Oral β-blocker (metoprolol, 20 or 40 mg) was administered 1 hour before the examination to achieve the target heart rate of 65 beats/min. A maximum dose of 12.5 mg of landiolol (Corebeta; Ono Pharmaceutical) was given intravenously if the heart rate was >65 beats/min in the CT room.^8^ All patients received 0.6 mg of sublingual nitroglycerin (Nitropen; Nippon Kayaku) before imaging. Patients received 18.0 mg iodine/kg/s of iomeprol (Iomeron 350; Eisai) for 12 seconds, followed by a 30-mL saline flush. The tube voltage was 100 kVp. The tube current was determined using automatic exposure control with a maximum of 900 mA. The target SD was 21 and reconstructed using a convolution kernel of FC04 with MBIR (Forward projected model-based Iterative Reconstruction SoluTion cardiac standard; Canon Medical Systems) for patients in the first half of the study. The target SD was increased to 26 and reconstructed using SR-DLR (Precise IQ Engine cardiac standard; Canon Medical Systems) in the second half. The reconstructed section thickness and increment were 0.50 mm and 0.25 mm, respectively. This change in institutional protocol was determined based on a phantom study to reduce the tube current while improving the spatial resolution.^7^ Images were transferred to a workstation for postprocessing (Synapse Vincent, version 6.7; Fujifilm Medical).

### Objective image analysis

Measurements were performed by a cardiovascular radiologist (N.T., with 15 years of experience) and a cardiovascular radiation technician (H.S., with 13 years of experience) who were blinded to the reconstruction method. Image noise at the aortic root, signal-to-noise ratio (SNR), and contrast-to-noise ratio (CNR) at the left main coronary artery trunk and proximal right coronary artery was quantified according to previously reported methods.^4^ The mean values of the 2 observers were used for analysis. Details are described in the Supplementary Methods.

### Subjective image analysis

Analyses were performed by a cardiovascular radiologist (N.T.) and a cardiovascular radiation technician (H.S.) who were blinded to the reconstruction methods. The reviewers scored the images independently using a 4-point quality score (1 = poor, 4 = excellent), according to the criteria defined in previous study for noise, blooming of calcification or stent, border conspicuity, and overall image quality.^9^ Analyses were performed on a per-patient and per-vessel basis. When scores differed between the 2 readers, the final score was determined by consensus.

### Coronary stenosis analysis

Measurements were performed by a cardiovascular radiologist (N.T.) and a cardiologist (D.T., with 5 years of experience) who were blinded to coronary angiography results. Any disagreements were discussed until consensus was reached. Stenosis was visually graded from 1 to 5: 1, 0% to 24% stenosis; 2, 25% to 49% stenosis; 3, 50% to 69% stenosis; 4, 70% to 99% stenosis; and 5, occluded. Significant stenosis was defined as stenosis of 50% or greater. Evaluations were performed on a per-vessel basis.

### Coronary angiography analysis

Invasive coronary angiography was performed according to a standard clinical practice. Coronary angiography images were visually evaluated on a per-vessel basis by 2 cardiologists (S.O. and S.K., with 28 and 23 years of experience, respectively) who were blinded to the clinical information of patients in a joint reading. Stenosis was visually graded from 1 to 5 in a similar manner to the coronary CT angiography analysis. Significant stenosis was defined as stenosis of 50% or greater.

### Statistical analysis

Continuous variables are shown as means ± SDs and categorical variables as frequencies with percentages, unless otherwise described. Student *t*-test was used to compare continuous variables. The Fisher exact test and Wilcoxon rank sum test were used to compare categorical variables and skewed variables, respectively. We adjusted the multiple vessels per patient. Interobserver agreement for subjective image quality was calculated using Cohen κ statistic, which was interpreted as poor (<0.20), fair (0.21-0.40), moderate (0.41-0.60), good (0.61-0.80), very good (0.81-0.90), or excellent (≥0.91). Intraclass correlation coefficient (ICC) was used to investigate interobserver variability for continuous variables.

Receiver operating characteristic curve analysis was used to compare the diagnostic value of coronary CT angiography to detect significant stenosis at coronary angiography. Subanalysis was performed based on the coronary artery calcium score and the presence of stents. Severe calcification was defined as calcium score >400.

The results of the post hoc power analysis showed that the achieved power was 78% to detect a difference in per-patient subjective overall image quality between MBIR and SR-DLR groups (α = .05).

Differences in AUC values were assessed using the DeLong method using the logistic regression analysis adjusted for multiple vessels per patient. Wilcoxon rank sum test adjusted for multiple vessels per patient, and calculation of ICCs were performed using R software (version 4.0.2; R Foundation for Statistical Computing). The remaining statistical analyses were performed using JMP software (version 17.0.0; SAS institute). A *P* value < .05 was considered to indicate a statistically significant difference.

## Results

### Patient characteristics and scan parameters

The study included 52 patients (mean age, 68 years ± 10 [SD]; 41 men) (Table 1). Mean heart rate during the scan was 57 beats/min ± 7 (SD), and all patients had diagnostic image quality. More than half of the patients had hypertension, dyslipidemia, and a smoking history. Five patients (10%) had a history of stent implantation. We found no evidence of differences in age, body mass index, cardiac risk factors, and Agatston score between the MBIR and SR-DLR groups.

**Table 1.** Patient demographics and scanning parameters.

	All patients	MBIR group	SR-DLR group	*P*
No. of patients	52	25	27	
Men/women	41/11 (79%/21%)	21/4 (84%/16%)	20/7 (74%/26%)	.50
Age (y)	68 ± 10	68 ± 11	69 ± 10	.92
Body mass index (kg/m^2^)	24.0 ± 3.5	23.4 ± 2.8	24.6 ± 4.1	.24
Cardiac risk factors				
Hypertension	34 (65%)	18 (72%)	16 (56%)	.39
Diabetes mellitus	17 (33%)	7 (28%)	10 (37%)	.56
Dyslipidemia	33 (63%)	16 (64%)	17 (63%)	1.0
Smoking, current or past	8/20 (15%/38%)	6/6 (24%/24%)	2/14 (7%/52%)	.07
Family history	9 (17%)	5 (20%)	4 (15%)	.72
After stent implantation	5 (10%)	1 (4%)	4 (15%)	.35
Agatston score	414 (137, 842)	407 (127, 730)	605 (137, 1507)	.55
Heart rate (beats/min)	57 ± 7	57 ± 8	57 ± 6	.77
Contrast medium (mL)	43 ± 9	42 ± 10	43 ± 9	.75
Tube current (mA)^b^	900 (695, 900)	900 (895, 900)	890 (680, 900)	.03^a^
Dose length product (mGy cm)^b^	203 (167, 244)	214 (180, 246)	196 (152, 219)	.17
Effective dose (mSv)^b^	2.8 (2.3, 3.4)	3.0 (2.5, 3.4)	2.7 (2.1, 3.1)	.17

Unless otherwise noted, values are expressed as number of patients, with percentages in parentheses or mean ± SD.

Abbreviations: MBIR = model-based iterative reconstruction, SR-DLR = super-resolution deep learning reconstruction.

a Statistically significant, *P* < .05.

b Values are expressed as median with IQR in parentheses.

**Figure 2.**
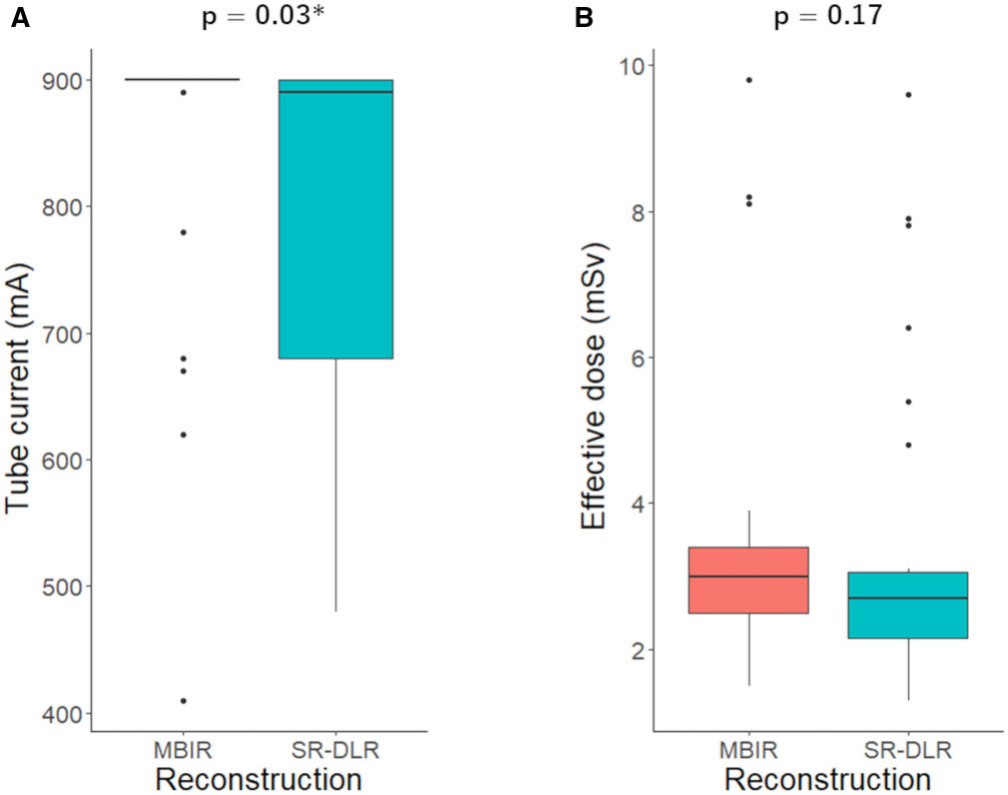
Comparison of tube current (A) and effective dose (B) reconstructed with MBIR and SR-DLR. Tube current of the SR-DLR group was less than that of the MBIR group (*P* = .03). MBIR = model-based iterative reconstruction, SR-DLR = super-resolution deep learning reconstruction.

The median tube current of the MBIR group was larger than that of the SR-DLR group (median [IQR], 900 mA [895, 900] vs. 890 mA [680, 900], *P* = .03) (Table 1, Figure 2). We found no evidence of a difference in radiation dose (median [IQR], 3.0 mSv [2.5, 3.4] vs. 2.7 mSv [2.1, 3.1], *P* = .17) and amount of contrast medium used (42 mL ± 10 vs. 43 mL ± 9, *P* = .75) between the MBIR and SR-DLR groups.

### Objective image analysis

**Figure 3.**
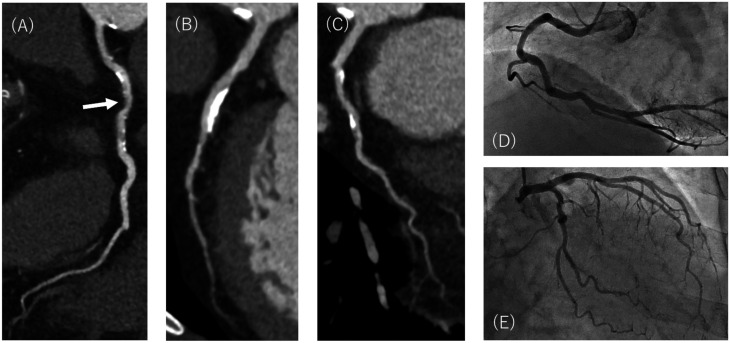
CT angiography images of (A) right coronary, (B) left anterior descending, and (C) left circumflex arteries in a 75-year-old female with atypical chest pain reconstructed with model-based iterative reconstruction. Middle segment of the right coronary artery was diagnosed as 50% to 69% stenosis partly from image noise (arrow). (D, E) Catheter-based conventional coronary angiography demonstrates no significant stenosis in all arteries.

**Figure 4.**
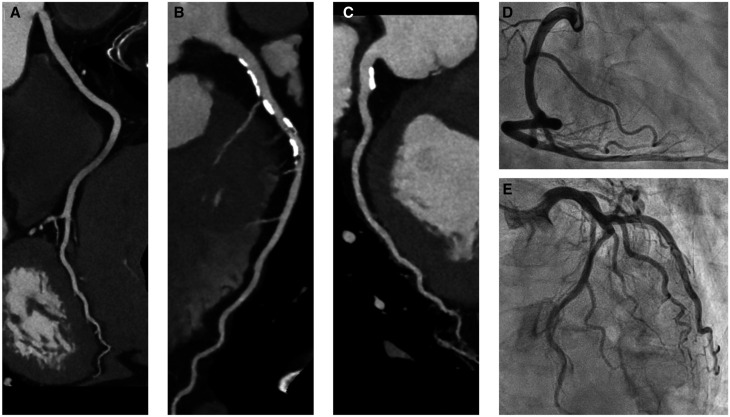
CT angiography images of (A) right coronary, (B) left anterior descending, and (C) left circumflex arteries in a 65-year-old male with atypical chest pain reconstructed with super-resolution deep learning reconstruction. Although the left anterior descending artery showed severe calcification, the degree of stenosis could be diagnosed as mild. (D, E) Catheter-based conventional coronary angiography demonstrates mild stenosis in the left anterior descending artery and no stenosis in other arteries.

We found no evidence of a difference in CT number of left main coronary artery and proximal right coronary artery (Table 2). Noise at the aortic root in the SR-DLR group was less than that in the MBIR group (14.6 HU ± 1.3 vs. 22.7 HU ± 4.4, *P* < .001). Therefore, both SNR of the left main coronary artery (31.2 ± 7.8 vs. 18.9 ± 4.6, *P* < .001) and proximal right coronary artery (36.5 ± 8.7 vs. 21.8 ± 5.3, *P* < .001) and CNR of the left main coronary artery (37.0 ± 8.5 vs. 22.1 ± 4.6, *P* < .001) and proximal right coronary artery (36.5 ± 8.7 vs. 21.8 ± 5.3, *P* < .001) were higher in the SR-DLR group than in the MBIR group (Figures 3 and 4).

**Table 2.** Objective image analysis.

Parameter	MBIR group	SR-DLR group	*P*
	(*n* = 25)	(*n* = 27)	
Noise at aortic root (HU)	22.7 ± 4.4	14.6 ± 1.3	<.001^a^
Left main coronary artery			
CT number (HU)	420 ± 89	453 ± 107	.88
Signal-to-noise ratio	18.9 ± 4.6	31.2 ± 7.8	<.001^a^
Contrast-to-noise ratio	22.1 ± 4.6	37.0 ± 8.5	<.001^a^
Right coronary artery			
CT number (HU)	410 ± 91	454 ± 111	.13
Signal-to-noise ratio	18.5 ± 4.8	31.3 ± 8.0	<.001^a^
Contrast-to-noise ratio	21.8 ± 5.3	36.5 ± 8.7	<.001^a^

Values are expressed as mean ± SD.

Abbreviations: HU = Hounsfield unit, MBIR = model-based iterative reconstruction, SR-DLR = super-resolution deep learning reconstruction.

a Statistically significant, *P* < .05.

### Subjective image analysis

On a per-patient analysis, image quality in the SR-DLR group was better than in the MBIR group in terms of noise (median [IQR], 4 [4, 4] vs 3 [3, 4], *P* < .001), border conspicuity (median [IQR], 4 [3, 4] vs 3 [3, 4], *P* = .01), and overall quality (median [IQR], 4 [3, 4] vs 3 [3, 3], *P* = .006) (Table 3, Figure S1). We found no evidence of difference in blooming and motion between the groups.

**Table 3.** Subjective image analysis.

Parameter	MBIR group	SR-DLR group	*P*
	(*n* = 25)	(*n* = 27)	
Per-patient analysis			
Noise	3 (3, 4)	4 (4, 4)	<.001^a^
Blooming	3 (2, 3)	3 (3, 3)	.53
Border conspicuity	3 (3, 4)	4 (3, 4)	.01^a^
Motion	4 (3, 4)	4 (3, 4)	.93
Overall	3 (3, 3)	4 (3, 4)	.006^a^
Left main coronary artery			
Noise	4 (3, 4)	4 (4, 4)	.002^a^
Blooming	4 (3, 4)	4 (3, 4)	.91
Border conspicuity	4 (4, 4)	4 (4, 4)	.34
Motion	4 (4, 4)	4 (4, 4)	.96
Overall	4 (3, 4)	4 (4, 4)	.03^a^
Left anterior descending artery			
Noise	4 (3, 4)	4 (4, 4)	.002^a^
Blooming	3 (3, 3)	3 (3, 3)	.80
Border conspicuity	4 (3, 4)	4 (3, 4)	.10
Motion	4 (4, 4)	4 (4, 4)	.43
Overall	3 (3, 4)	4 (3, 4)	.04^a^
Left circumflex artery			
Noise	4 (3, 4)	4 (4, 4)	<.001^a^
Blooming	3 (3, 4)	3 (3, 4)	.89
Border conspicuity	3 (3, 4)	4 (3, 4)	.006^a^
Motion	4 (4, 4)	4 (4, 4)	.17
Overall	3 (3, 4)	4 (3, 4)	.005^a^
Right coronary artery			
Noise	3 (3, 4)	4 (4, 4)	<.001^a^
Blooming	3 (3, 4)	3 (3, 4)	.79
Border conspicuity	3 (3, 4)	4 (4, 4)	.006^a^
Motion	4 (3.5, 4)	4 (4, 4)	.45
Overall	3 (3, 4)	4 (4, 4)	<.001^a^

Values are expressed as median with IQR in parentheses.

Abbreviations: MBIR = model-based iterative reconstruction, SR-DLR = super-resolution deep learning reconstruction.

a Statistically significant, *P* < .05.

When analyzed per-vessel, noise and overall quality of the SR-DLR group were better than those of the MBIR group in the left main coronary artery (Table 3, Figure S2) and left anterior descending artery (Table 3, Figure S3). Noise, border conspicuity, and overall quality of the SR-DLR group were better than those of the MBIR group in the left circumflex artery (Table 3, Figure S4) and right coronary artery (Table 3, Figure S5).

### Coronary stenosis analysis

**Figure 5.**
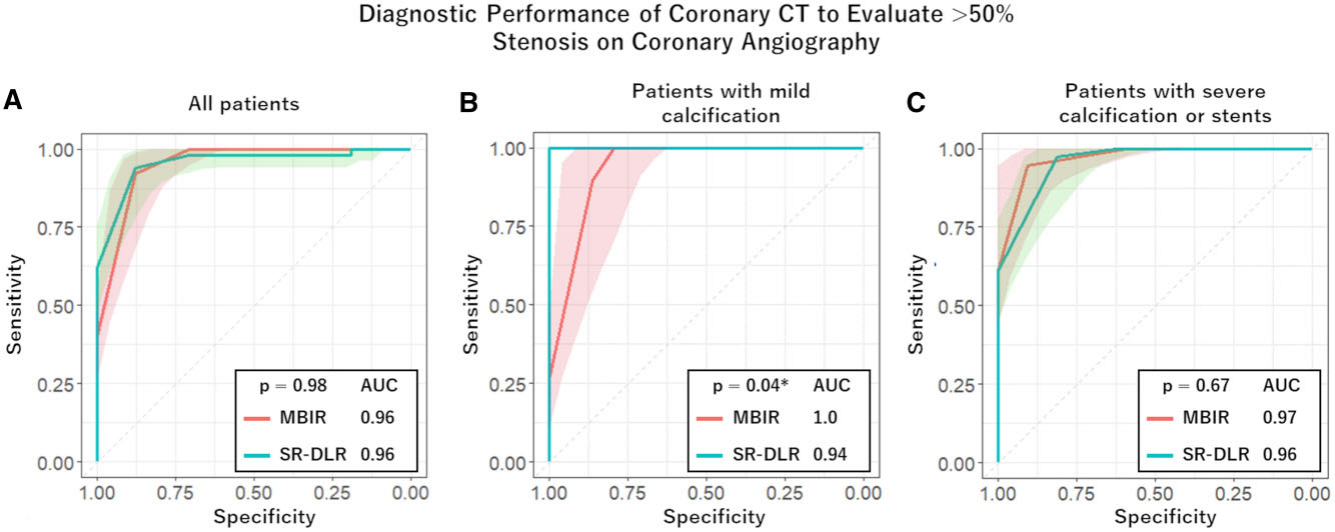
Comparison of receiver operating characteristic curves evaluating performance of coronary CT angiography in diagnosing >50% stenosis in all patients (A), patients with mild calcification (B), and patients with severe calcification or stents (C). Shaded areas represent the 95% CI. MBIR = model-based iterative reconstruction, SR-DLR = super-resolution deep learning reconstruction. *Statistically significant, *P* < .05.

In the MBIR group, 39 of 100 vessels (39%) in 22 of 25 patients (88%) showed significant stenosis on coronary angiography. In the SR-DLR group, 50 of 108 vessels (46%) in 24 of 27 patients (89%) showed significant stenosis on coronary angiography. Receiver operating characteristic analysis for evaluating the performance of each reconstruction algorithm in diagnosing significant coronary stenosis on coronary angiography showed no difference in AUC of MBIR (0.96; 95% CI, 0.92-0.98) and SR-DLR (0.96; 95% CI, 0.92-0.99; *P* = .98) (Figure 5A). The AUC of SR-DLR (1.0; 95% CI, 1.0-1.0) was significantly higher than that of MBIR (0.94; 95% CI, 0.88-0.996; *P* = .04) in patients with mild calcification (Figure 5B). The AUC of SR-DLR (0.96; 95% CI, 0.93-0.996) and MBIR (0.97; 95% CI, 0.94-1.0; *P* = .67) in patients with severe calcification or stents showed no significant difference (Figure 5C).

### Interobserver analysis

Interobserver ICC for noise, SNR, and CNR ranged from 0.95 (95% CI, 0.91-0.97) to 0.97 (95% CI, 0.95-0.98), indicating good agreement (Table 4). SNR (0.78; 95% CI, 0.11-1.45; *P* = .02) and CNR (0.94; 95% CI, 0.10–1.78; .03) of left main coronary artery and SNR of right coronary artery (0.64; 95% CI, 0.00-1.28; *P* = .048) showed significant difference between the observers. We found no significant difference in noise and CNR of right coronary artery between the observers.

**Table 4.** Interobserver agreement of objective analysis.

	ICC	Mean difference	*P*
Noise	0.95 (0.91-0.97)	−0.32 (−0.78 to 0.14)	.17
SNR of left main coronary artery	0.96 (0.93-0.98)	0.78 (0.11-1.45)	.02^a^
CNR of left main coronary artery	0.95 (0.92-0.97)	0.94 (0.10-1.78)	.03^a^
SNR of right coronary artery	0.97 (0.95-0.98)	0.64 (0.00-1.28)	.048^a^
CNR of right coronary artery	0.95 (0.91-0.97)	0.62 (−0.33 to 1.57)	.19

Values are expressed as mean with 95% CI in parentheses.

Abbreviations: CNR = contrast-to-noise ratio, ICC = intraclass correlation coefficient, SNR = signal-to-noise ratio.

a Statistically significant, *P* < .05.

Interobserver agreement on noise, blooming, border conspicuity, motion, and overall quality were moderate (κ  =  0.55), moderate (κ  =  0.51), good (κ  =  0.65), moderate (κ  =  0.53), and moderate (κ  =  0.57), respectively (Table S1).

## Discussion

The results of the present study suggest that compared with MBIR, SR-DLR could reduce image noise (*P* < .001) and increase SNR (*P* < .001) and CNR (*P* < .001) with reduced tube current settings. Subjective image quality of SR-DLR images showed better scores than MBIR images (*P* = .006) by improving noise (*P* < .001) and border conspicuity (*P* = .01). We found no significant difference in the diagnostic performance to detect morphological significant stenosis on coronary CT angiography between the MBIR and SR-DLR groups (*P* = .98), although certainty of these conclusions is limited because of our small cohort size. To the best of our knowledge, this is the first study to evaluate image quality and diagnostic performance of coronary CT angiography using SR-DLR with reduced tube current.

Compared with other reconstruction algorithms, such as filtered back projection, iterative reconstruction, and normal-resolution DLR, SR-DLR improves spatial resolution while reducing image noise.^7^ This benefits coronary CT angiography, especially in patients with stents and coronary calcification.^4–6^ A previous study showed that subjective image quality of stents using SR-DLR was superior to that using MBIR by reducing the strut thickness.^6^ This is in line with the present study in that border conspicuity of SR-DLR images was better than MBIR images, but differs in that SR-DLR did not improve blooming of calcification. Spatial resolution assessed by task-based transfer function differs by the strength of iterative reconstruction.^7^ The 10% and 50% spatial frequency value of task-based transfer function decreases when a stronger algorithm is used, resulting in reduction of spatial resolution. Although not described in the previous study,^6^ there might be a difference in the strength of the iterative reconstruction used with the present study.

Evaluation of noncalcified coronary plaques is important to predict future cardiovascular events, because vulnerable plaques tend to have low CT values.^10^ A previous study investigating subjective scores on coronary artery plaques showed that SR-DLR could better depict noncalcified coronary plaques than other reconstruction methods, including MBIR.^4^ Other studies focusing on low-contrast lesion detectability have been performed in abdominal diseases. Regardless of the type of DLR, DLR could better depict low contrast lesions compared with iterative reconstruction by reducing image noise and increasing image sharpness.^11–13^ These results are consistent with the present study in that border conspicuity improved using SR-DLR than MBIR.

We found no significant difference in diagnostic performance of SR-DLR and MBIR to detect morphological significant stenosis in coronary CT angiography. This is different from a previous study that showed that SR-DLR could improve diagnostic accuracy and specificity than MBIR.^6^ However, this study was limited to stent analysis, and segments without stents were excluded from analysis. Difference in diagnostic performance was not found in a subgroup of patients with severe calcification or stents in this study. Our small cohort size could also have accounted for the absence of a significant difference in diagnostic performance between the two reconstruction techniques.

Coronary CT angiography reconstructed with MBIR has an excellent diagnostic performance to detect coronary stenosis by coronary CT angiography, with an accuracy greater than 85%^14^^,^^15^; therefore, the diagnostic performance to detect morphological coronary stenosis in general might not improve using DLR. However, there might be the prospect for DLR to improve the accuracy of functional stenosis assessed by CT fractional flow reserve because the precision of computational fluid dynamics depends on the reproducibility of coronary structure. Although a previous study failed to show the superiority of normal-resolution DLR to MBIR in diagnosing functionally significant stenosis,^16^ SR-DLR might be a breakthrough to further improve the diagnostic performance.

Radiation dose of coronary CT angiography has decreased with the introduction of novel reconstruction methods. Of note, coronary CT angiography with submillisievert radiation dose has become available using MBIR.^15^^,^^17^^,^^18^ Although we set the noise level of the automatic exposure control higher when SR-DLR was introduced, the reduction of the tube current was not as we initially expected. This occurred because tube current was determined at the caudal level of the heart where the liver overlaps. It is possible to further reduce the radiation dose if we set the tube current manually, but not without drawbacks. A phantom study showed that SR-DLR could improve spatial resolution and image noise not only when using low tube current, but also when using standard tube current.^7^ In other words, excessive reduction of radiation dose might cancel out the benefits of SR-DLR. In fact, ultra-low-dose abdominal CT with DLR did not reach the image quality of standard-dose CT with hybrid iterative reconstruction.^19^

This study had the following limitations. First, this was a single-center study using a single CT system and reconstruction algorithm. The results might differ when DLR of other vendors are used. Second, the effect of DLR in diagnosing functional coronary stenosis have not been tested. Comparing the CT fractional flow reserve using different reconstruction methods would answer this question. Third, the mean body mass index was lower than a typical Western population. The tube current in this study might be suboptimal in obese patients. Fourth, we did not compare images reconstructed with normal-resolution DLR in this study. Finally, the small cohort size limits any conclusions that can be drawn about the lack of observed differences, including diagnostic performance and radiation dose.

In conclusion, the present exploratory analysis indicates that SR-DLR could improve objective and subjective image quality with lower tube current settings than MBIR with similar diagnostic performance to diagnose morphological coronary stenosis in coronary CT angiography. Further research with larger sample sizes and using algorithms of different vendors may help add to the growing body of evidence favoring the use of SR-DLR in coronary CT angiography. Focused studies for identifying the optimal balance between the image quality and radiation dose are also warranted.

## Supplementary Material

umae001_Supplementary_Data

## Data Availability

Data generated or analyzed during the study are available from the corresponding author by request.
