# Supplementary material for: Super-resolution deep learning reconstruction to improve image quality of coronary CT angiography
Source: Radiol Adv. 2024 Mar 19;1(1):umae001. doi: 10.1093/radadv/umae001 (PMC12428329; doi:10.1093/radadv/umae001)
Supplement: umae001_Supplementary_Data [file umae001_Supplementary_Data.zip › umae001_Supplementary_Data/Supplementary Table S4.docx]

**Supplementary Table S4:** Series ratings by sequence sorted by percent rated "Equal".

| **Name:** | **Equal** | **Reduced** | **Not Diagnostic** | **Rescanned/Excluded** | **All** | **Percent Equal (%)** |
| --- | --- | --- | --- | --- | --- | --- |
| ***Sequence type:* PMC** | | | | | | |
| Ax DW-EPI | 31 | 1 | 1 | 0 | 33 | 93.9 |
| Cor T2w-PROP 2mm | 22 | 4 | 0 | 0 | 26 | 84.6 |
| Ax T2w-PROP 2mm | 16 | 3 | 0 | 0 | 19 | 84.2 |
| Cor T2-FLAIR-PROP | 46 | 4 | 1 | 6 | 57 | 80.7 |
| Ax 2D SWI-EPI | 26 | 3 | 1 | 3 | 33 | 78.8 |
| Ax T1-FLAIR-PROP | 35 | 6 | 0 | 6 | 47 | 74.5 |
| *All PMC* | 230 | 35 | 4 | 42 | 311 | 74.0 |
| Sag T2w-PROP 4mm | 20 | 7 | 0 | 1 | 28 | 71.4 |
| Ax T2w-PROP 4mm | 26 | 5 | 1 | 5 | 37 | 70.3 |
| Sag 3D T1w-SPGR | 8 | 2 | 0 | 21 | 31 | 25.8 |
| ***Sequence type:* NeuroMix modules** | | | | | | |
| DW-EPI | 50 | 6 | 1 | 8 | 65 | 76.9 |
| 3D SWI-EPI | 49 | 5 | 3 | 8 | 65 | 75.4 |
| T1-FLAIR-PROP | 35 | 6 | 1 | 6 | 48 | 72.9 |
| Sag 3D T1w-EPI | 47 | 5 | 5 | 8 | 65 | 72.3 |
| T2-FLAIR-SSFSE | 47 | 9 | 1 | 8 | 65 | 72.3 |
| *All NeuroMix* | 322 | 57 | 18 | 56 | 453 | 71.1 |
| T2*-EPI | 44 | 12 | 1 | 8 | 65 | 67.7 |
| T2w-SSFSE | 44 | 9 | 4 | 8 | 65 | 67.7 |
| T1-FLAIR-EPI | 6 | 5 | 2 | 2 | 15 | 40.0 |
