# Supplementary material for: Super-resolution deep learning reconstruction to improve image quality of coronary CT angiography
Source: Radiol Adv. 2024 Mar 19;1(1):umae001. doi: 10.1093/radadv/umae001 (PMC12428329; doi:10.1093/radadv/umae001)
Supplement: umae001_Supplementary_Data [file umae001_Supplementary_Data.zip › umae001_Supplementary_Data/umae001_Supplementary_Data.docx]

**Super-Resolution Deep Learning Reconstruction to Improve Image Quality of Coronary CT Angiography**

**Article type:** Original research

**Summary Statement**

Super-resolution deep learning reconstruction improved both objective and subjective image quality over model-based iterative reconstruction in the diagnosis of coronary artery stenosis, with similar diagnostic performance and reduced tube current.

**Key Results**

- Super-resolution deep learning reconstruction reduced image noise compared to model-based iterative reconstruction with reduced tube current (14.6 HU ± 1.3 vs. 22.7 HU ± 4.4, p <0.001).
- The overall subjective image quality using super-resolution deep learning reconstruction was better than that using model-based iterative reconstruction (median [IQR], 4 [3, 4] vs. 3 [3, 3], p = 0.006).
- The area under the receiver operating characteristic curve in diagnosing coronary stenosis using super-resolution deep learning reconstruction (0.96; 95% CI: 0.92–0.99) and model-based iterative reconstruction (0.96; 95% CI: 0.92–0.98; p = 0.98) seem comparable.

**Supplemental Methods**

**Objective Image Analysis**

Image noise was defined as the standard deviation of CT number at the aortic root. The CT number of the coronary artery lumen was quantified by placing the largest possible region of interest at the left main coronary artery trunk and proximal right coronary artery, carefully avoiding the inclusion of the vessel wall and plaque. The CT number of the epicardial fat around each analyzed coronary lumen was also measured. The signal-to-noise ratio was calculated by dividing the CT number of the coronary lumens by the image noise. The contrast-to-noise ratio was calculated by dividing the difference of CT number between the coronary lumens and the surrounding epicardial fat by the image noise.

**Supplemental Figures**


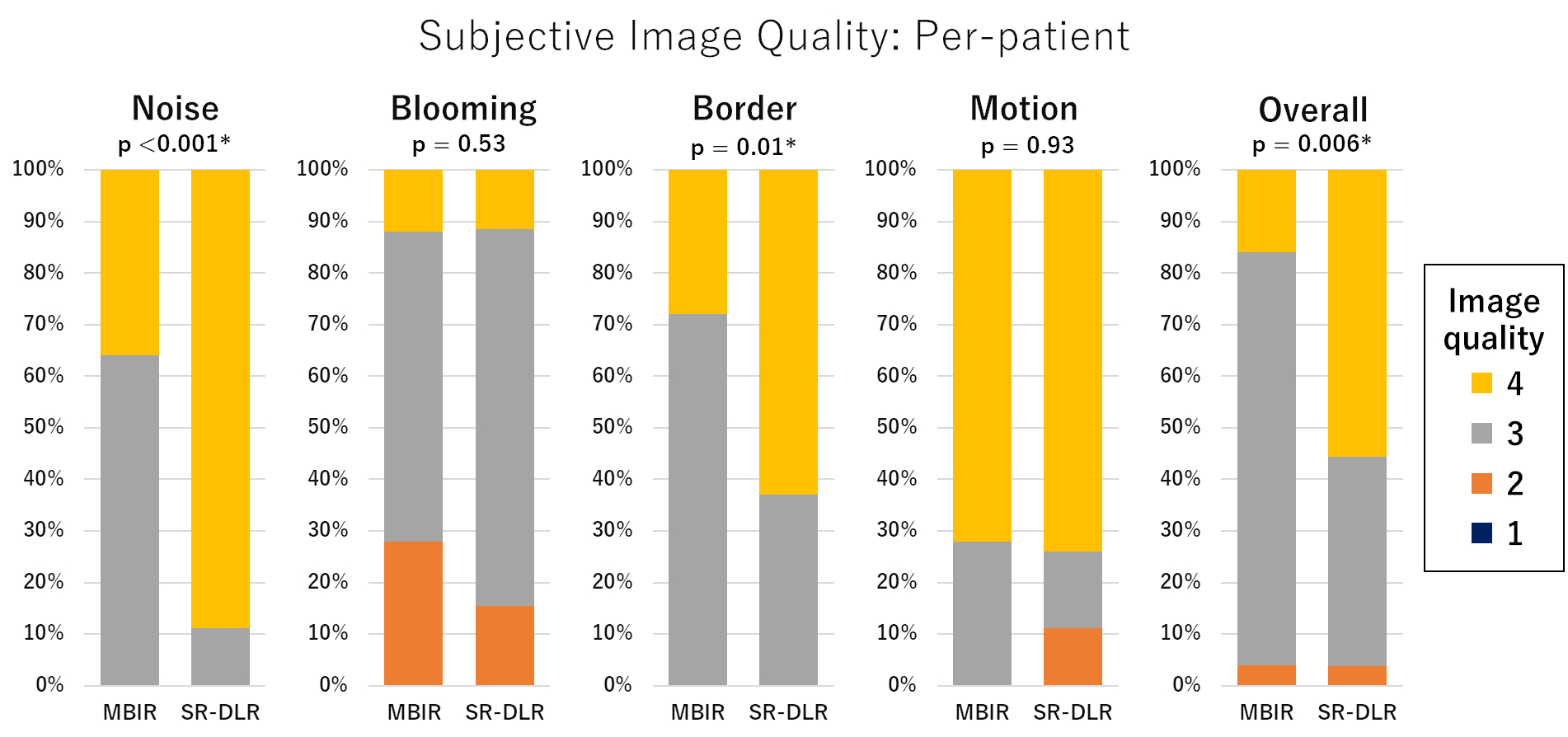


**Figure S1:** Comparison of subjective image quality between MBIR and SR-DLR images in per-patient analysis. The image quality score of SR-DLR was better than MBIR in noise, border conspicuity, and overall image quality (p <0.05).

MBIR = model-based iterative reconstruction, SR-DLR = super-resolution deep learning reconstruction


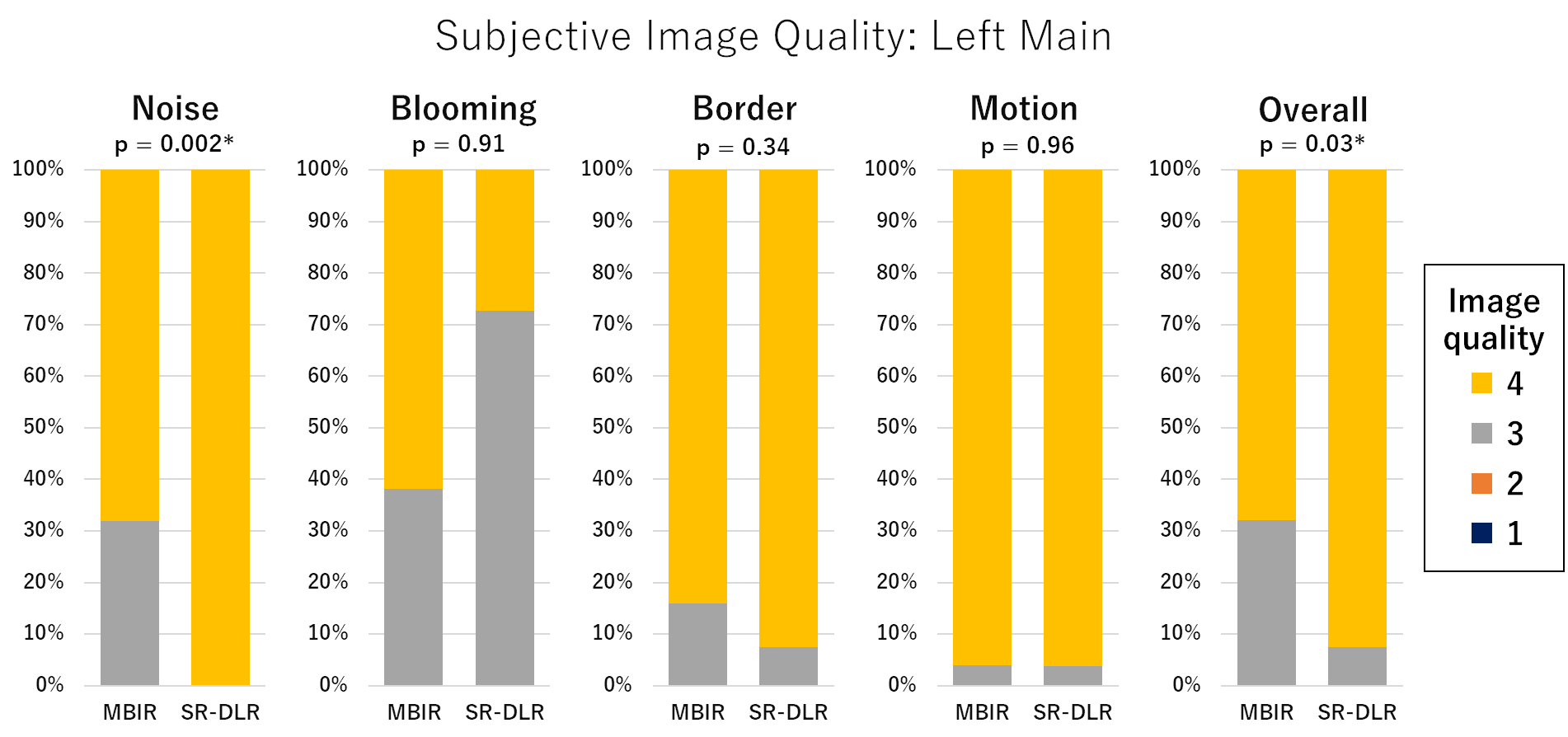


**Figure S2:** Comparison of subjective image quality between MBIR and SR-DLR images in the left main coronary artery. The image quality score of SR-DLR was better than MBIR in noise, and overall image quality (p <0.05).

MBIR = model-based iterative reconstruction, SR-DLR = super-resolution deep learning reconstruction


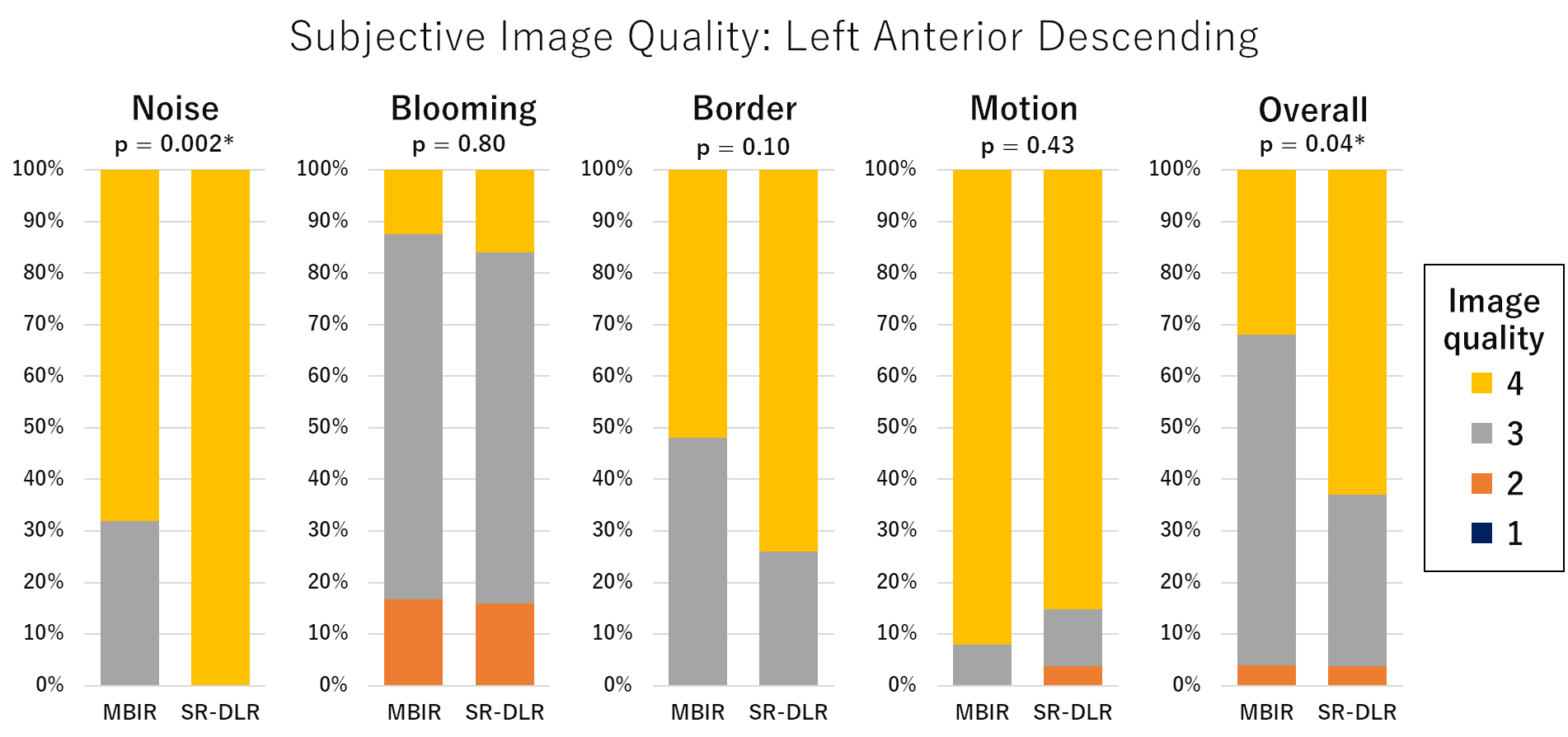


**Figure S3:** Comparison of subjective image quality between MBIR and SR-DLR images in the left anterior descending artery. The image quality score of SR-DLR was better than MBIR in noise, and overall image quality (p <0.05).

MBIR = model-based iterative reconstruction, SR-DLR = super-resolution deep learning reconstruction


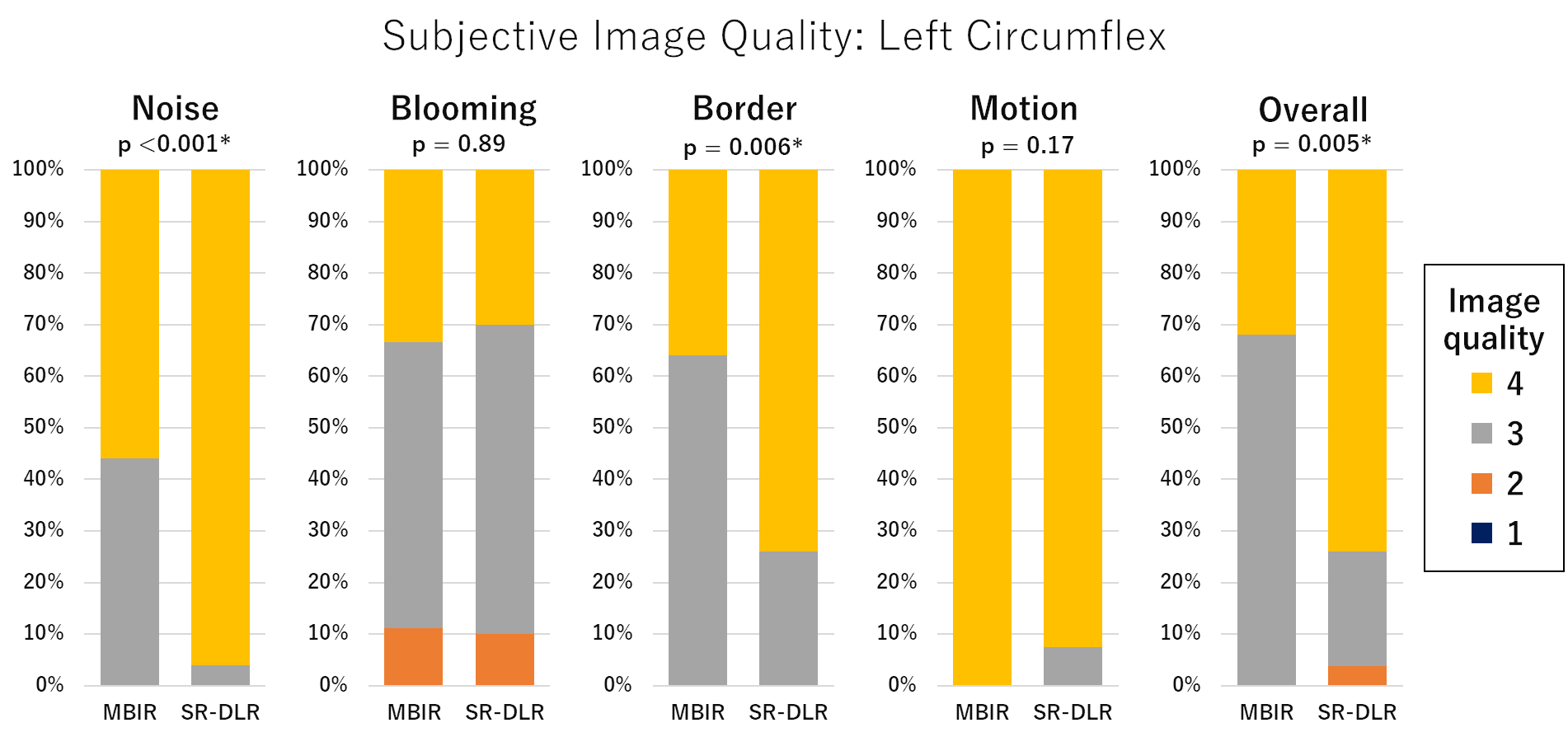


**Figure S4:** Comparison of subjective image quality between MBIR and SR-DLR images in the left circumflex artery. The image quality score of SR-DLR was better than MBIR in noise, border conspicuity, and overall image quality (p <0.05).

MBIR = model-based iterative reconstruction, SR-DLR = super-resolution deep learning reconstruction


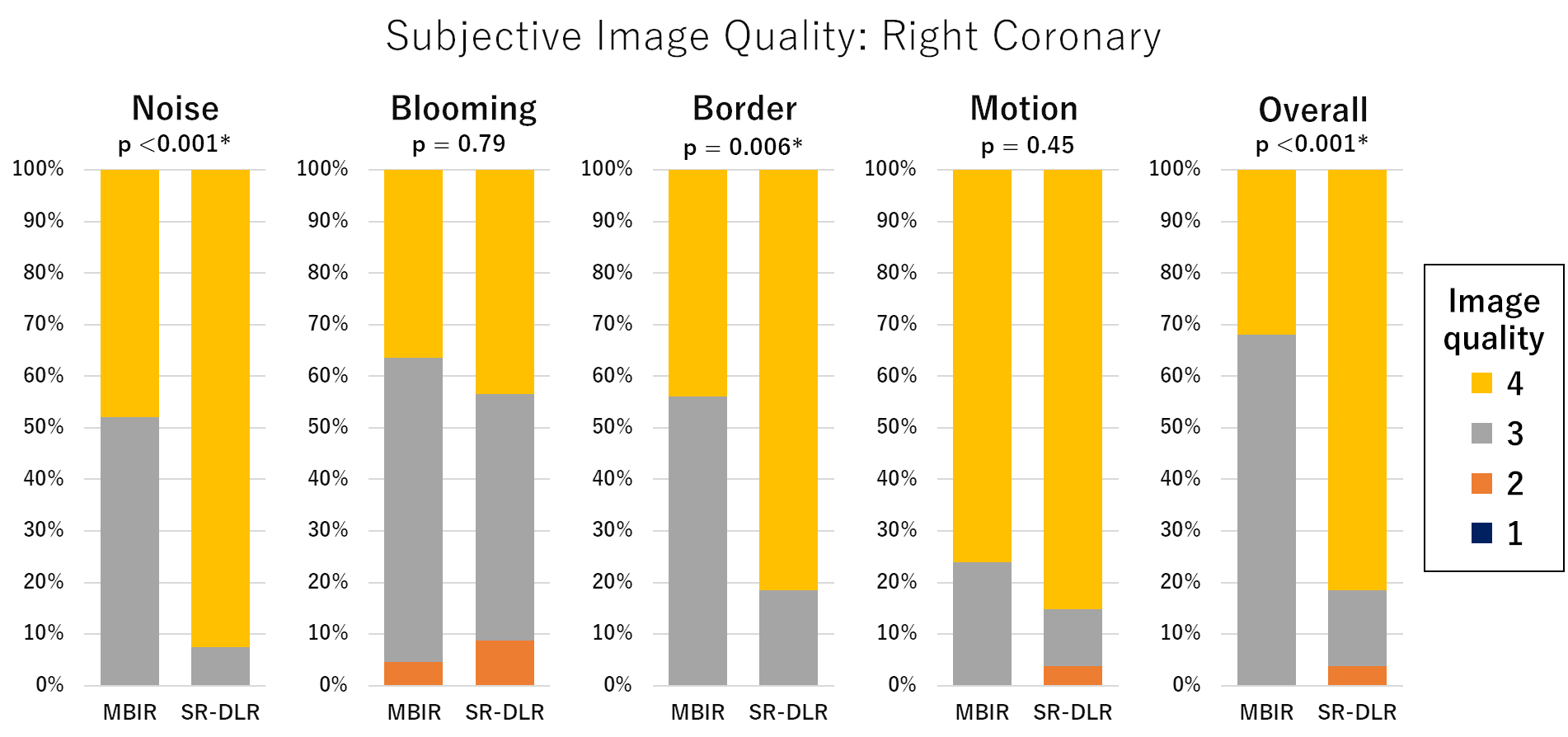


**Figure S5:** Comparison of subjective image quality between MBIR and SR-DLR images in the right coronary artery. The image quality score of SR-DLR was better than MBIR in noise, border conspicuity, and overall image quality (p <0.05).

MBIR = model-based iterative reconstruction, SR-DLR = super-resolution deep learning reconstruction

**Supplemental Table**

**Table S1** Interobserver Agreement of Subjective Analysis

|  | Cohen κ statistic |
| --- | --- |
| Noise | 0.55 (0.40–0.69) |
| Blooming | 0.51 (0.39–0.63) |
| Border conspicuity | 0.65 (0.53–0.76) |
| Motion | 0.53 (0.35–0.71) |
| Overall | 0.57 (0.46–0.69) |

Note.— Values are expressed as mean with 95% CI in parentheses.
